# Association Between Surrounding Greenness and Schizophrenia: A Taiwanese Cohort Study

**DOI:** 10.3390/ijerph16081415

**Published:** 2019-04-19

**Authors:** Hao-Ting Chang, Chih-Da Wu, Wen-Chi Pan, Shih-Chun Candice Lung, Huey-Jen Su

**Affiliations:** 1 Department of Environmental and Occupational Health, National Cheng Kung University, Tainan 70101, Taiwan; s78041018@mail.ncku.edu.tw; 2 Department of Geomatics, National Cheng Kung University, Tainan 70101, Taiwan; chidawu@mail.ncku.edu.tw; 3 Institute of Environmental and Occupational Health Sciences, National Yang-Ming University, Taipei 11221, Taiwan; wenchipan@post.harvard.edu; 4 Research Center for Environmental Changes, Academia Sinica, Taipei 11529, Taiwan; sclung@rcec.sinica.edu.tw; 5 Department of Atmospheric Sciences, National Taiwan University, Taipei 10617, Taiwan; 6 Institute of Environmental Health, School of Public Health, National Taiwan University, Taipei 10617, Taiwan

**Keywords:** schizophrenia, normalized difference vegetation index, greenness, incidence, cohort study

## Abstract

This study aims to investigate the association between surrounding greenness and schizophrenia incidence in Taiwan. Data of 869,484 individuals without a history of schizophrenia were included from the Longitudinal Health Insurance Database from 2000 through 2010 for analysis. The diagnoses of schizophrenia were based on ICD-9 codes. Greenness exposure was assessed using the satellite-based normalized difference vegetation index, assuming individuals lived near the hospital they most often visited for common cold during the study period. Cox proportional hazards models were applied to assess the association between greenness exposure and schizophrenia incidence after adjustments were made for the potential confounders. A total of 5,069 schizophrenia cases were newly diagnosed during the study period. A negative significant (*p* < 0.05) association found using 2000-m buffer distances (distance of a moderately paced 20-min walk) in the whole Taiwan island, cities, and metropolitan areas. The results of the stratified analysis based on sex and health insurance rate suggested surrounding greenness has approximately equal effects of reducing the risk of schizophrenia, regardless of sex or financial status. In conclusion, our findings suggest that more surrounding greenness may reduce the risk of schizophrenia.

## 1. Introduction

Mental disorders are a health condition characterized by alterations in thinking, mood, and behavior associated with distress or impaired functioning. Mental disorders may lead to a host of severe consequences including disability, pain, or death. Mental disorders comprise a broad range of conditions with various symptoms. However, they are generally characterized by a combination of abnormal thoughts, emotions, behaviors, and relationships with others [1]. According to the National Institute of Mental Health, nearly one in five United States adults lives with a mental illness; this number accounted for more than 44.7 million people in 2016 [2]. Mental disorders are the leading cause of disability worldwide. Over 300 million people experience depression, equivalent to 4.4% of the world’s population, and more than 260 million people experience anxiety disorder, representing 3.6% of the population worldwide [3]. Furthermore, the WHO reported that mental disorders account for nearly 10.9% of years lived with disability [3]. The resulting disease burden of mental illness is among the highest of all diseases. Schizophrenia is a chronic, severe, and disabling disorder that has affected human beings throughout history [4]. Schizophrenia is one of the 15 leading causes of disability worldwide [5]. The peak age of onset for schizophrenia is between 15 and 30 years (15 to 25 years in men and 20 to 30 years in women) [6]. The symptoms of schizophrenia fall into three broad categories, namely positive symptoms, negative symptoms, and cognitive symptoms [7]. The positive symptoms include hallucinations, delusions, thought disorders (unusual or dysfunctional ways of thinking), and movement disorders (agitated body movements). The negative symptoms include flat affect (reduced expression of emotions via facial expressions or voice tones), reduced feelings of pleasure in everyday life, difficulty beginning and sustaining activities, and reduced speaking. The cognitive symptoms include poor executive functioning (the ability to understand information and use it to make decisions), trouble focusing or paying attention, and problems with working memory (the ability to use information immediately after learning it). Schizophrenia can be caused by several factors, such as genetic and environmental factors, exposure to viruses, malnutrition before birth, problems during birth, and psychosocial factors [7]. The possible biological mechanisms that play a role in schizophrenia included an imbalance in the complex, interrelated chemical reactions of the brain involving neurotransmitters, dopamine, and glutamate, among others [7].

Urbanization is one of the major factors contributing to mental disorders (including schizophrenia). Urbanization is a process that leads to the loss of green spaces and the growth of cities. Urban studies have revealed that urbanization affects mental health through the influence of increased stressors and factors such as overcrowded, polluted, and warming environments; high levels of violence; and limited social support [8,9]. Approximately 55% of the global population currently lives in urban areas, and it is estimated that two of every three people will live in cities in 2050 [10].

From the literature reviews, studies have demonstrated that exposure to areas with higher amounts of distributed community vegetation has numerous health benefits, including higher levels of physical activity, lower levels of obesity, fewer cardiovascular diseases, and lower overall mortality [11,12]. Moreover, trees and vegetation can lower surface and air temperatures through evapotranspiration and the provision of shade [13]. This cooling effect also brings direct and indirect benefits to human health and prevents unnecessary deaths during heatwaves [14]. Researchers have increasingly examined the link between exposure to greenness and mental health outcomes [11,12,15]. However, no study has examined the relationship between greenness and mental health in Asia at a national scale with a broad diversity of greenness.

Therefore, this study analyzed the association of greenness and schizophrenia in Taiwan from 2000 to 2010 based on the Longitudinal Health Insurance Database (LHID) developed by the National Health Insurance (NHI) system. Furthermore, the association was assessed in cities or metropolitan areas. This is the first study to investigate the linkage between surrounding greenness and mental health in Asia using a retrospective cohort study design with a considerable number of participants (one million).

## 2. Materials and Methods 

### 2.1. Study Area

Taiwan is an island located on the Tropic of Cancer; its climate regions consist of the northern and central subtropical and southern tropical regions. Because of the variety of its climate regions, Taiwan is the habitat for numerous species. Taiwan also lies in the circum-Pacific seismic zone at the intersection of the Yangtze Plate, Okinawa Plate, and Philippine Mobile Belt. This location causes the height above sea level to increase continuously and produces earthquakes. The total area of Taiwan is 36,197 km^2^ [16], and the geography is characterized by rugged mountains running from north to south in the eastern two-thirds of the island and flat plains in the western third. The western region is also home to most of Taiwan’s population. In Taiwan, the second half of the 20th century is called “Taiwan’s economic miracle” because of the rapid economic growth and industrialization [17]. Between 1952 and 1982, the economic growth was on average 8.7% per year; between 1983 and 1986, it was 6.9% per year. The gross national product increased by 360% between 1965 and 1986. However, accompanying the economic development, the prevalence of common mental disorders doubled from 11.5% in 1990 to 23.8% in 2010 [18].

### 2.2. Databases

#### 2.2.1. Remotely Sensing Greenness Data

**Figure 1:**
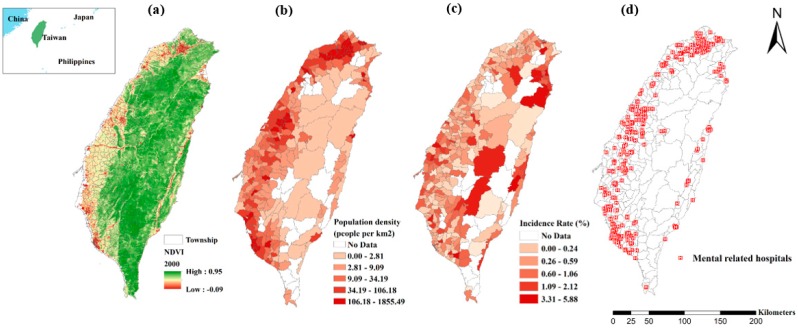
Databases displayed in the location of Taiwan. (**a**) The spatial variability of greenness within the whole island based on the MODIS NDVI (Normalized Difference Vegetation Index) image of 2000. (**b**) The township scale of population density (**c**) The township scale of incidence rate from 2000 to 2010. (**d**) The location of mental related hospitals in Taiwan.

We used the normalized difference vegetation index (NDVI) as the index of surrounding greenness throughout our analysis. The NDVI data were extracted using the global moderate-resolution imaging spectroradiometer maintained by the National Aeronautics and Space Administration (NASA) and United States Geological Survey. The NDVI is a spectrum-based greenness index for measuring and monitoring plant growth (vigor), vegetation cover, and biomass production from multispectral satellite data. Healthy vegetation that is growing vigorously has low red-light reflectance and high near-infrared reflectance; thus, it shows high NDVI values. Unhealthy or sparse vegetation reflects more visible light and less near-infrared light. This relative algorithm produces output values in the range of −1.0 to 1.0. Positive NDVI values increase with the amount of healthy green vegetation. NDVI values near zero and decreasing negative values indicate nonvegetative features, such as barren surfaces (rock and soil), water, snow, ice, and clouds. This database provides NDVI information every 16 days at 250-m spatial resolution as a gridded product in the sinusoidal projection. We applied the annual average of NDVI in 2000 as the long-term exposure to surrounding greenness of participants, because NDVI in Taiwan did not show annual variation between 2000 and 2010. Spatially averaged NDVI values with circular buffer from distances of 1000 to 2000 m (10–20 min of moderately paced walking) and a 250-m interval were calculated to represent the greenness exposure for each participant. The geographical location of Taiwan covered by the NDVI spatial illustration in 2000 is presented in Figure 1a.

#### 2.2.2. LHID

We used the LHID, a subset of the NHI, as our main data source to assess the association between NDVI and schizophrenia. The NHI database was established in 1995 and is a single-payer and compulsory social insurance system that regulates the disbursement of health care funds and guarantees equal access to health care for all citizens. As of 2014, 99.9% of the citizens in Taiwan (approximately 23,340,000 citizens) were enrolled in this program. The LHID contains the medical information of a million randomly sampled individuals from the NHI database. The original claims and a registry of the beneficiaries that includes demographic characteristics, inpatient and ambulatory care, diagnostic codes, catastrophic illness certificates, medical expenditures, operations, prescriptions, examinations, procedures, hospital levels, and medical divisions are recorded in this database for research purposes [19,20]. After validation, the individuals included in the LHID and those enrolled in the original Taiwan NHI did not differ particularly in terms of age, sex, mean health insurance rate, or distribution in townships [20]. The identification of schizophrenia incidence from 2000 to 2010 in the LHID was based on the International Classification of Diseases, Ninth Revision (ICD-9) codes 295.0–295.9. We adopted the 1996 to 1999 period as a wash-out period; patients who had schizophrenia during this period were masked out from the analysis. Participants residing in off-shore islands such as Kinmen and Matsu were excluded. In total, 869,484 individuals without a history of schizophrenia were recruited for our analysis. The LHID provides the hospital details for each participant’s medical claim, instead of providing their residential address for confidentiality reasons. We assumed all patients lived near the hospital they most often visited for a common cold (ICD-9 code: 460 or 465) during the study period. A point of interest (POI) database, which records the distribution of more than a million landmarks in Taiwan, was used to identify the geolocation of the participants, as well as their level of exposure to greenness. In general, we used the location of the most frequently visited medical services during the study period as a proxy for residential address. Circular buffers surrounding the selected hospital or clinic were subsequently generated, and the average of the NDVI values within different buffer ranges was calculated accordingly to represent the participants’ greenness exposure. The township-scale population density during the study period is displayed in Figure 1b. The township-scale incidence rate during the study period is displayed in Figure 1c. Demographic and socioeconomic data, namely age, sex, health insurance rate (proxy for personal financial status), and classification of the insured were acquired from the LHID. Six categories were used to classify the insured. The first category included civil servants, volunteer military personnel, public office holders, private school teachers, employees of public or private owned enterprises and organizations, employers self-employed, independent professionals, and technical specialists. The second category included trade union members and foreign crew members. The third category included members of farming, fishing, and irrigation associations. The fourth category included military conscripts, alternative military service personnel, military school students on scholarships, widows or widowers of deceased military personnel on pensions, and inmates. The fifth category included low-income households. The sixth category included veterans and their dependents and other individuals. The NHI premiums for the individuals in category 1, 2, and 3 are calculated based on the monthly income they report to the NHI administration. The premiums for the individuals in category 4, 5, and 6 are based on the average premium of the people enrolled in category 1, 2, and 3 [21]. The study protocol was reviewed and approved by the National Cheng Kung University Governance Framework for Human Research Ethics (No. 104-009), and the research had been conducted according to principles of Declaration of Helsinki.

#### 2.2.3. Meteorological Information

Monthly weather records were acquired from 333 monitoring stations of the Central Weather Bureau located in Taiwan. The spatial average of monthly mean temperature, and total precipitation, at a township resolution was estimated using an ordinary kriging interpolation method with a spherical model. The obtained cross-validated *R*^2^ value ranges for temperature, and precipitation were 0.62–0.76, and 0.45–0.82 respectively. These values demonstrated that estimates obtained from a kriging interpolation could serve to approximate the spatial distribution pattern of meteorological factors in Taiwan.

#### 2.2.4. Mental-Related Information

The mental-related hospital information was extracted from the point of interest (POI) database in Taiwan. The mental-related hospitals included medical centers, regional hospitals, teaching hospitals, general hospitals, district hospitals, mental health centers, united municipal hospitals, and psychiatric hospitals. The total number of mental-related hospitals was 758. The locations of mental-related hospitals in Taiwan are illustrated in Figure 1d.

### 2.3. Statistical Analysis

We hypothesized that exposure to greenness has a positive effect on mental health. With the retrospective cohort study design, the Cox proportional hazards model was used to estimate the association of long-term exposure to surrounding greenness with the risk of schizophrenia [22,23]. The variables in the model were considered because research has proven them correlated with the NDVI, and collinearity was avoided by employing the Spearman correlation analysis. The inclusion criteria for the variables selection were a correlation greater than 0.6 between independent variables and references proving that one of the variables correlates with the NDVI. To examine the relationship between NDVI and schizophrenia, we analyzed the hazard ratio (HR) of schizophrenia incidence within different degrees of greenness from 2000 m buffers in comparison with the lowest greenness (for the lowest greenness, the NDVI was less than 0.26). The categorical method was based on previous research [24]. Adjustments were made using the representations of a range of well-established factors obtained from the LHID, including age (0 to 15, 15 to 30, older than 30 years), sex, health insurance rate (stratifying the data into two groups based on a previous study [25]: Less than NT$20,000 and more than NT$20,000), and classification of the insured. Metrological factors such as mean temperature, total precipitation, and relative humidity were also considered for model adjustment because previous studies have suggested that weather conditions affect mental status [26,27]. A *p* value of 0.05 was selected for statistical significance. ArcGIS (version 10.4.1; Environmental Systems Research Institute Inc., Redlands, CA) and SAS 9.4 (Cary, NC, U.S.A.) were used for the analyses.

### 2.4. Sensitivity Test and Stratified Analysis

To examine the nonlinear relationship between NDVI and schizophrenia, we used two approaches to conduct the sensitivity test. The first approach was to use different buffer sizes of greenness, a continuous variable, within 1000-m and 2000-m buffer distances and with a 250-m interval (10–20 min of moderately paced walking) [28]. In a previous study, particulate matter was reported as a major environmental factor affecting mental health [29], but the earliest time of PM_2.5_ (particulate matter ≤2.5 μm in diameter) monitoring was 2005. Although complete PM_2.5_ monitoring data could not be collected in the study period, the PM_10_ (particulate matter ≤10 μm in diameter) level was higher than that of PM_2.5_; therefore, PM_10_ was considered a surrogate. Consequently, the 2000-m buffer distance with the addition of PM_10_ was analyzed to demonstrate the robustness of the observed association and define the outcome of the sensitivity test. The PM_10_ data were acquired from 76 air quality monitoring stations of the Environmental Protection Administration, Executive Yuan, Republic of China (Taiwan). The spatial average of monthly PM_10_ was estimated using an ordinary kriging interpolation method with a spherical model. The obtained cross-validated R^2^ value range was 0.61–0.84. These values demonstrated that the estimates obtained from a kriging interpolation could be used to approximate the spatial distribution pattern of air pollution in Taiwan. The second approach was restricted to participants in main city areas, including Taipei City and Kaohsiung City, or in metropolitan areas, including the Taipei and combined Taipei and Kaohsiung metropolitan areas, to assess the effects of greenness in urban areas. Moreover, we used sex and health insurance rate as stratification variables, dividing the data into four groups, namely men and women and poorer (less than NT$20,000) and richer (more than NT$20,000) individuals. We calculated the *p* value for the HR to determine the statistical significance between the NDVI and stratified variables of schizophrenia risk.

## 3. Results

### 3.1. Descriptive Statistics

In Table 1, participants are stratified by their NDVI values into 10 stratifications to observe the demographic distribution in the assorted greenness levels. The mean NDVI values for the greenness stratifications from lowest to highest were 0.22, 0.27, 0.30, 0.33, 0.39, 0.47, 0.53, 0.58, 0.64, and 0.74. The highest mean health insurance rate was NT$13,912.55 in the fourth stratification (10th–24th percentile of NDVI [NDVI from 0.31 to 0.35]), and the lowest was NT$12,348.53 in the eighth stratification (90th–94th percentile of NDVI (NDVI from 0.56 to 0.60)). A higher proportion of men lived in greener places. The highest proportion of the age classification was 15 to 30 years old, the peak age of onset for schizophrenia, in the 25th–49th percentile (24.05%), and the lowest was in stratification below the first percentile (15.90%). The highest proportion of people with health insurance rates of less than NT$20,000 was in the 25th–49th percentile (53.60%), and the lowest was in the stratification above the 99th percentile (43.08%). The fifth stratification (25th–49th percentile of NDVI (NDVI from 0.35 to 0.43)) included the lowest sociodemographic characteristics, but it did not include the highest schizophrenia incidence rate (0.57%), implying that environmental factors play a role in the incidence of schizophrenia.

### 3.2. Model Analysis and Sensitivity Test

Table 2 depicts the association between schizophrenia and the categorical NDVI with a 2000-m buffer distance in Taiwan. An HR less than 1 indicates that the population received beneficial effects from greenness exposure. An HR of more than 1 signified a higher hazard of schizophrenia from greenness exposure. After adjustments were made for demographic, socioeconomic, and environmental factors, the coefficients of surrounding greenness revealed a significantly negative association (*p* < 0.05) with the incidence of schizophrenia compared with the lowest NDVI classification; the most negative association was in the highest NDVI classification (HR = 0.37, 95% confidence interval (CI) = (0.25–0.55)). In addition, in the sensitivity analysis (Table 3), the HRs obtained from all of the models were smaller than the one, namely the NDVI as a continuous variable from 1000- to 2000-m buffer distances with the 250-m interval and subgroup analysis in cities and metropolitan areas. This result indicated that exposure to surrounding greenness had a protective effect on individuals, thus reducing the risk of schizophrenia. Specifically, it appeared that the protective trend increased with the buffer size. Moreover, the protective effect existed not only in cities proper (HR = 0.22, 95% CI = (0.06–0.81)) but also in metropolitan areas (HR = 0.46, 95% CI = (0.25–0.85)).

**Figure 2:**
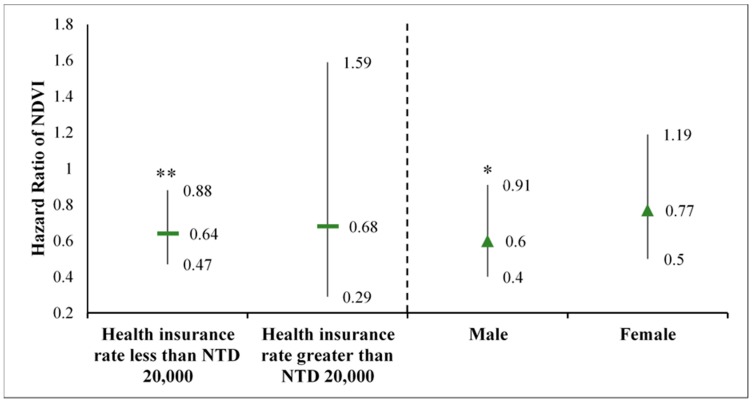
Stratified analysis for the hazard ratio of NDVI (estimates with 95% confidence interval) according to sex and health insurance rate less (poorer) than NTD 20,000 and health insurance rate greater than NTD 20,000 (richer). Significance level: * indicates *p* value < 0.05, ** indicates *p* value < 0.01, and *** indicates *p* value < 0.001.

Figure 2 presents the results of the stratified analysis based on two stratification variables: health insurance rate (proxy of personal financial status) and sex. Protection benefits from surrounding greenness were observed against schizophrenia incidence. For the group with health insurance rates of less than NT$20,000 and the male group, the HR of the NDVI was less than 1 and significant (*p* < 0.05), suggesting that greenness beneficial effects concerning schizophrenia incidence.

## 4. Discussion

The lowest sociodemographic characteristic was observed in the fifth greenness exposure category (the proportion of the health insurance rate less than NT$20,000 = 53.60%, Table 1). Although various factors affect the risk of developing or triggering schizophrenia and personal financial status is one of the dominant factors [30], no highest schizophrenia incidence rate (0.57%) was found in that stratification. Therefore, the environmental factors of greenness might play a crucial role in incidence. This hypothesis was demonstrated through the main model (Table 2), the sensitivity model (Table 3), and the stratified model (Figure 2). First, in the main model, the protective effect of greenness exposure was observed; specifically, the strongest protective effect was noted in the highest NDVI classification of the main model. Consistent findings were reported in recent studies [31]. In the sensitivity model, beneficial effects were associated with an increase in buffer size and depended on whether it was in the city or in a metropolitan area. As previously discussed, a significant negative correlation was observed between the greenness exposure and self-rated propensity to psychiatric morbidity in a 1-km and 3-km radius around the postal code coordinates for each individual’s address [32]. Another reason was that larger buffers were more likely to exist in greener covers, which provided people with more opportunities to participate in activities [28]. Moreover, a similar effect in different administrative divisions, that is, rural areas, provincial towns, provincial cities, capital suburbs, and capital centers was reported in a recent study [31]. Furthermore, the Taipei metropolitan area exhibited a stronger protective effect than the combined Taipei and Kaohsiung metropolitan areas. This may be caused by the superior economy [33] and medical support [34] of the Taipei metropolitan area. In addition, a stable result between greenness exposure and mental health outcomes was demonstrated when annual PM_10_ levels were considered [29].

In the stratified model, we associated a decreasing risk of having schizophrenia with not only different health insurance levels but also the participants’ sex. Although not all of the results were significant in the stratified group, the average trend revealed a protective effect. Similar results according to the different socioeconomic status were demonstrated by related studies [35]. Similarly, higher levels of greenness were associated with even greater mental health benefits in low-income neighborhoods. As investigated in a previous study, a similar association between participants’ sex, nature connectedness, and internalized mental health symptoms was observed [36].

The results of the Cox models indicate that exposure to surrounding greenness protects people against schizophrenia. The health benefits of greenness have been widely discussed, but few studies have addressed the medical mechanisms of that phenomenon [37,38]. Based on the limited number of studies discussing this, the potential mechanisms can be condensed into “relaxation” and “immune system improvement.” Encounters with nearby nature help alleviate mental fatigue by relaxing and restoring the mind. Parks and green spaces in built-up environments are ideal settings for cognitive respite; they encourage social interaction, reduce stress through exercise or conversation, and provide calming settings. Creating quality landscape and vegetation in and around work and study places is a valuable investment. Both visual and physical access to green spaces helps restore the mind’s ability to focus. Such a setting can improve work and school performance and assist in alleviating mental stress and illness [39,40]. Moreover, some studies have stated that the phytoncide derived from plants during forest bathing trips substantially increase the number of natural killer cells, perforins, granzymes, and granulysin, thus strengthening the immune system and improving physical and mental health [37,41]. In addition, some studies have reported a correlation between forest bathing and anxiety alleviation [42,43] A recent application of that theory was introduced through healing gardens as part of the wider complex at the Villa di Salute Clinic for mental disorders. Specifically, a treatment for schizophrenia was developed in Turin (Piedmont, Italy) to promote the health and well-being of patients and provide them with comfort and relief from illness [44].

This study also revealed that men had a higher HR for schizophrenia than women, and people between 15 and 30 years old had the highest risk of schizophrenia compared with other age classifications. These results are similar to the findings in past studies [6,45].

The economic decline might explain the rapid degradation of mental health in Taiwan [18]. The unemployment rate doubled from 2.74% to 5.74% in the last decade, and the economic growth rate drastically decreased from 6.42% in 2000 to −1.57% in 2009 [46]. The stagnating economy considerably affects mental health in Taiwan. The low-income household category is the most at risk for schizophrenia compared with other categories of insurants. A consistent finding is that the highest proportion of people with schizophrenia is located in low- or middle-income regions, particularly among individuals, farmers, retirees, and less educated, unemployed, unmarried, or divorced individuals [47].

Strong associations between meteorological factors (temperature, relative humidity, and precipitation) and schizophrenia were observed in this study. Although a reverse association with temperature was observed in a previous study [48], the time scale might have been the cause. Previous studies have used either daily or monthly temperature reports. However, in this study, the temperature considered was the yearly temperature. Therefore, the effect of temperature was attenuated, and the range was minimized. The temperature ranged from 17 to 23 °C in this study, a range typically within the range of thermal comfort; therefore, a beneficial effect could exist. The potential reason for the negative association between relative humidity and schizophrenia is the fact that trees and vegetation can modify the environmental humidity through evapotranspiration [13]. More greenness implies more relative humidity, which in turn reduces the risk of schizophrenia. The positive association between precipitation and schizophrenia was consistent with a previous finding [49].

This is the first retrospective cohort study to examine the health benefits from greenness exposure to combat schizophrenia. Physician-diagnosed information recorded in the NHI system for more than 800,000 individuals was employed for model analysis. Moreover, a remotely sensed greenness database established by NASA representing not only the spatial distribution but also the vigor of plants was adopted to quantify the long-term greenness exposure for each individual. Nevertheless, this study had some limitations. For example, the mailing address of each participant was masked by the database providers because of privacy policy. Instead, the location of the hospital visited for a common cold (because people usually visit the nearest hospital for minor illnesses) was used to determine participants’ exposure to surrounding greenness. For example, the mailing address of each participant was masked by the database providers because of their privacy policy. Instead, the location of the hospital visited for a common cold (because people usually visit the nearest hospital for minor illnesses) was used to determine participants’ exposure to surrounding greenness. This approach might also induce some bias. For example, in rural townships with limited medical services, the most frequently visited hospital or clinic might not be close to the participant’s residence. Any relocation events or trips abroad were not taken into account; therefore, the actual exposure during that period might not be precise.

Furthermore, the gene information and family history of participants were unknown. In 2013, research suggested the effects of single-nucleotide polymorphisms are much less relevant than environmental factors [50]. According to this finding, missing gene information and family history should not limit our conclusions regarding surrounding greenness and schizophrenia. We encourage further investigation to duplicate these analyses after the aforementioned limitations have been resolved.

## 5. Conclusions

This study used a national scale cohort database to examine the association between greenness and schizophrenia. The results revealed that people with higher exposure to greenness experience lower schizophrenia incidence. Given the economic aspects of the social and medical burden of schizophrenia and the quality of long-term care, greenness is a crucial element to consider in the planning of future urban areas and policy. For example, treatments at psychiatric hospitals could be designed according to scientific evidence provided by environmental psychology and neuroscience, such as encouraging walks and exercise in green spaces. Further research is required in additional locations to determine the effects of greenness on different ethnic groups. We suggest that future studies duplicate these analyses in different countries to assess the effects of geographical differences on the benefits of green spaces.

## Figures and Tables

**Table 1:** Baseline characteristics of study population stratified by greenness exposure (N = 869,484).

Greenness Exposure	<1st (N = 4006)	1st–4th (N = 34,627)	5th–9th (N = 41,591)	10th–24th (N = 144,006)	25th–49th (N = 210,814)	50th–74th (N = 212,808)	75th–89th (N = 135,532)	90th–94th (N = 42,666)	95th–99th (N = 34,789)	>99th (N = 8645)	
	Mean (SD)	Mean (SD)	Mean (SD)	Mean (SD)	Mean (SD)	Mean (SD)	Mean (SD)	Mean (SD)	Mean (SD)	Mean (SD)	*p* *
NDVI	0.22 (0.05)	0.27 (0.01)	0.30 (0.01)	0.33 (0.02)	0.39 (0.02)	0.47 (0.02)	0.53 (0.02)	0.58 (0.01)	0.64 (0.02)	0.74 (0.02)	<0.001
Health insurance rate	12,812.28 (15,286.60)	13,153.26 (15,363.66)	13,128.03 (15,929.97)	13,912.55 (16,607.39)	12,400.93 (15,081.04)	12,836.53 (14,831.04)	12,728.42 (14,284.15)	12,348.53 (13,373.57)	12,686.02 (12,681.59)	12,535.24 (12,000.73)	<0.001
	No. (%)	No. (%)	No. (%)	No. (%)	No. (%)	No. (%)	No. (%)	No. (%)	No. (%)	No. (%)	*p* *
Sex											
Male	2127 (53.1)	16,964 (48.99)	20,767 (49.93)	69,309 (48.13)	104,891 (49.76)	108,603 (51.03)	69,190 (51.05)	22,138 (51.89)	18,376 (52.82)	4649 (53.78)	<0.001
Female	1879 (46.9)	17,663 (51.01)	20,824 (50.07)	74,697 (51.87)	105,923 (50.24)	104,205 (48.97)	66,342 (48.95)	20,528 (48.11)	16,413 (47.18)	3996 (46.22)	--
Age (years)											
0–15	160 (3.99)	1895 (5.47)	2389 (5.74)	8993 (6.24)	16,054 (7.62)	15,825 (7.44)	10,000 (7.38)	3099 (7.26)	2073 (5.96)	471 (5.45)	<0.001
15–30	637 (15.90)	7629 (22.03)	8085 (19.44)	32,601 (22.64)	50,691 (24.05)	50,386 (23.68)	30,907 (22.80)	9702 (22.74)	7351 (21.13)	1770 (20.47)	--
30 +	3209 (80.1)	25,103 (72.50)	31,117 (74.82)	102,412 (71.12)	144,069 (68.34)	146,597 (68.89)	94,625 (69.82)	29,865 (70.00)	25,365 (72.91)	6404 (74.08)	--
Health insurance rate											
NTD 0–20,000	2102 (52.47)	17,716 (51.16)	22,103 (53.14)	74,952 (52.05)	113,003 (53.60)	107,866 (50.69)	66,872 (49.34)	20,587 (48.25)	15,524 (44.62)	3724 (43.08)	<0.001
NTD 20,000 +	1904 (47.53)	16,911 (48.84)	19,488 (46.86)	69,054 (47.95)	97,811 (46.40)	104,942 (49.31)	68,660 (50.66)	22,079 (51.75)	19,265 (55.38)	4921 (56.92)	--
Psychological status											
Incidence cases of schizophrenia	50 (1.25)	174 (0.50)	337 (0.81)	721 (0.50)	1211 (0.57)	1209 (0.57)	858 (0.63)	236 (0.55)	218 (0.63)	55 (0.64)	<0.001

* *p* values were based on the Wilcoxon rank-sum test for no classified NDVI and health insurance rate or Pearson’s chi-squared for sex, age, health insurance rate, psychological status incidence cases of schizophrenia. All statistical tests were two-sided. NDVI = Normalized Difference Vegetation Index, NTD = New Taiwan dollars.

**Table 2:** Hazard ratios (HR) and confidence interval (C.I.) for categorical NDVI from 2000 m buffers in cox models.

Variable	HR (95% C.I.)	*p*-Value
NDVI		
<1st (0.26)	1.00	
1st (0.26)–4th	0.44 (0.32, 0.61)	<0.001
5th (0.29)–9th	0.69 (0.51, 0.93)	0.01
10th (0.31)–24th	0.45 (0.34, 0.60)	<0.001
25th (0.35)–49th	0.47 (0.35, 0.62)	<0.001
50th (0.43)–74th	0.49 (0.37, 0.65)	<0.001
75th (0.50)–89th	0.49 (0.37, 0.65)	<0.001
90th (0.56)–94th	0.41 (0.30, 0.56)	<0.001
95th (0.60)–99th	0.41 (0.30, 0.55)	<0.001
≥99th (0.69)	0.37 (0.25, 0.55)	<0.001
Age (years)		
0–15	1	
15–30	3.90 (3.25, 4.67)	<0.001
>30	2.71 (2.27, 3.24)	<0.001
Sex		
Female	1	
Male	1.03 (0.98, 1.09)	0.29
Health insurance rate (NTD)		
≤20,000	1	
>20,000	0.77 (0.70, 0.85)	<0.001
Classification of the insured		
Category 1	1	
Category 2	1.14 (1.05, 1.24)	<0.01
Category 3	1.10 (1.00, 1.21)	0.05
Category 4	1.28 (0.78, 2.10)	0.32
Category 5	6.40 (5.47, 7.49)	<0.001
Category 6	2.42 (2.24, 2.61)	<0.001
Temperature (°C)	0.90 (0.88, 0.92)	<0.001
Relative humidity (%)	0.24 (0.18, 0.31)	<0.001
Precipitation (mm)	1.08 (1.06, 1.09)	<0.001

*p* value were two-sided. NDVI = Normalized Difference Vegetation Index; NTD = New Taiwan dollars.

**Table 3:** Hazard ratios (HR) and confidence interval (C.I.) for NDVI in cox models in sensitivity.

Model	No. of Participant (Case)	HR (95% C.I.)	*p*-Value
NDVI as a continuous variable			
NDVI from 1000 m buffers	869,249 (5065)	0.96 (0.70, 1.31)	0.79
NDVI from 1250 m buffers	869,484 (5069)	0.81 (0.60, 1.11)	0.19
NDVI from 1500 m buffers	869,484 (5069)	0.73 (0.54, 0.99)	0.04
NDVI from 1750 m buffers	869,484 (5069)	0.69 (0.51, 0.93)	0.02
NDVI from 2000 m buffers	869,484 (5069)	0.67 (0.50, 0.91)	0.01
NDVI from 2000 m buffers with addition of PM_10_	869,484 (5069)	0.68 (0.51, 0.92)	0.01
Subgroup analysis			
Taipei City	111,642 (690)	0.24 (0.04, 1.35)	0.11
Kaohsiung City	60,989 (365)	0.21 (0.03, 1.64)	0.14
Taipei and Kaohsiung City	172,631 (1055)	0.22 (0.06, 0.81)	0.02
Restricted to participants in Taipei metropolitan ^1^ area	257,302 (1389)	0.15 (0.06, 0.37)	<0.001
Restricted to participants in Taipei metropolitan ^1^ and Kaohsiung metropolitan ^2^ area	363,575 (2020)	0.46 (0.25, 0.85)	0.01

All models were adjusted for age, sex, health insurance rate, classification of the insured, temperature, relative humidity, and precipitation, except Subgroup analysis of Taipei City and Kaohsiung City, which was not considered relative humidity. ^1^ Taipei metropolitan area, namely Taipei City and New Taipei City, ^2^ Kaohsiung metropolitan area, namely Kaohsiung City and Kaohsiung County.
